# A High-Gain Circularly Polarized Magnetoelectric Dipole Antenna Array for Millimeter-Wave Applications

**DOI:** 10.3390/s25103046

**Published:** 2025-05-12

**Authors:** Jun Xiao, Jing Wu, Zihang Ye, Tongyu Ding, Chongzhi Han, Qiubo Ye

**Affiliations:** 1 School of Ocean Information Engineering, Jimei University, Xiamen 361000, China; xiaojun@jmu.edu.cn (J.X.); wujing2023@jmu.edu.cn (J.W.); 202021114019@jmu.edu.cn (Z.Y.); tyding@jmu.edu.cn (T.D.); 2 College of Information Science and Engineering, Hohai University, Changzhou 213200, China

**Keywords:** high gain, circularly polarized (CP), millimeter-wave (MMW), magnetoelectric dipole (ME-dipole), substrate-integrated waveguide (SIW), antenna array

## Abstract

A high-gain circularly polarized (CP) magnetoelectric dipole (ME-dipole) radiating element is demonstrated at a millimeter-wave (MMW) 5G band of 37–43.5 GHz. Each ME-dipole radiating element, consisting of two pairs of ring-shaped and L-shaped metal posts is excited by a cross-shaped substrate-integrated waveguide (SIW) coupling slot to achieve CP radiation. Through the use of all-metal radiating structures with a height of 3.4 mm, high-gain and high-efficiency radiation performances are achieved. For proof of concept, a 4 × 4 antenna array with a SIW feeding network is designed, fabricated, and measured. The measured impedance bandwidth of the proposed 4 × 4 CP antenna array is 19.2% from 33.9 to 41.1 GHz for |S11| ≤ −10 dB. The measured 3 db AR bandwidth is 10.3% from 37 to 41 GHz. The measured peak gain is 20.3 dBic at 41 GHz. The measured and simulated results are in good agreement.

## 1. Introduction

The increasing adoption of millimeter-wave (MMW) technology has sparked a surge in interest for broadband high-gain antennas across industry and academia. While the MMW band offers ample spectrum resources, it also presents significant propagation loss challenges. Consequently, high-gain antennas are crucial to mitigate this issue. Typically, antenna arrays comprising radiating elements and feeding networks are engineered to achieve high gain. Metallic waveguide-based antenna arrays are favored for their low-loss characteristics, yet their bulky profile, heavy weight, and high fabrication costs impede widespread implementation in MMW communications. However, in recent years, a planar waveguide structure known as substrate-integrated waveguide (SIW) has gained popularity in MMW applications due to its advantages of low loss and ease of integration [1].

Circularly polarized (CP) antennas have the ability of low attenuation in rain and snow and are not affected by Faraday effect compared to linearly polarized (LP) antennas. Various planar MMW CP antenna arrays have been extensively studied [2,3,4,5,6,7,8,9,10,11]. In addition to the low-loss feeding networks, high-gain and broadband antenna element is important to an antenna array.

Because of advantages of compact and simple structure, microstrip patch and slot antennas are widely adopted as the radiating element to compose a large array. However, microstrip patch and slot antennas have the drawbacks of narrow bandwidth, unstable radiating patterns, low radiation efficiency. The magnetoelectric dipole (ME-dipole) antenna was first proposed by Luk [12], which features the advantages of broad bandwidth, low back lobe level and stable radiation patterns. It can be a good candidate for composing broadband and high-gain MMW antenna arrays. Therefore, several MMW CP ME-dipole antennas have been reported in [13,14,15,16,17,18,19,20,21]. However, these CP ME-dipole antennas use microstrip patch as radiation structures, which leads to relatively low gains due to the dielectric losses.

In this paper, a wideband and high-gain SIW-fed CP ME-dipole antenna element and a 4 × 4 array are proposed in the MMW 5G band of 37–43.5 GHz. Each ME-dipole radiating element, consisting of two pairs of ring-shaped and L-shaped metal posts is excited by a cross-shaped SIW coupling slot. By the use of all-metal radiating structures with a height of 3.4 mm, high gain and high efficiency have been achieved. The simulated and measured results demonstrated that the proposed 4 × 4 antenna array achieves wideband, high gain, and stable radiation patterns, which indicates that the proposed array is a good candidate for MMW communication.

## 2. ME-Dipole Antenna Array Design

### 2.1. Proposed CP Antenna Element Design

**Figure 1:**
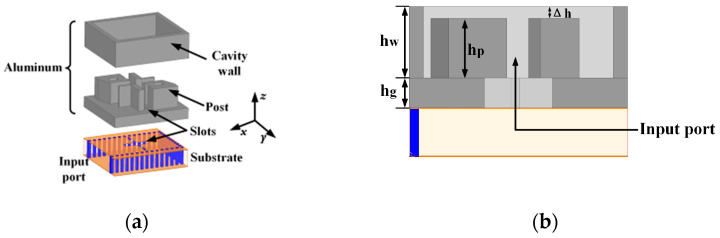

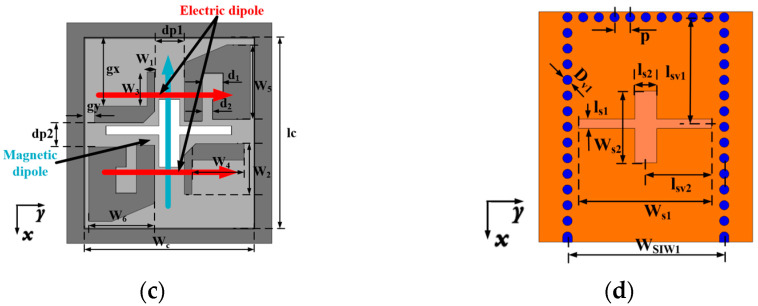
Geometry of the proposed CP antenna element. (**a**) 3D exploded view. (**b**) Side view. (**c**) Top view of the top aluminum plate. (**d**) Top view of the bottom substrate.

Figure 1 shows the geometry of the designed SIW-fed CP ME-dipole antenna element. This antenna element consists of a metallic radiating part and a bottom layer of dielectric substrate, where the substrate is Rogers 5880 (εr = 2.2, tanδ = 0.0009, Rogers Corporation, Chandler, AZ, USA) with a thickness of 1.575 mm. The proposed antenna is fed by a cross-shaped SIW slot. For the metallic radiating part, the surfaces of two pairs of metal posts form two pairs of electric dipoles. The metal posts are supported by a 1 mm ground plane, and the metal posts forming each pair of electric dipoles are located on either side of the SIW slot. A metal cavity is loaded around the four metal posts. The equivalent magnetic dipole is realized by the gap between the metal posts and the wall as well as the gap between the metal posts. The proposed ME-dipole is fed by a cross-shaped SIW slot which consists of a short slot and a long slot. Through the cross-shaped slot, the 3 dB AR bandwidth can be expanded. The optimized values of the final design parameters of the antenna element are tabulated in Table 1.

#### 2.1.1. Analysis of the Proposed CP ME-Dipole

**Figure 2:**
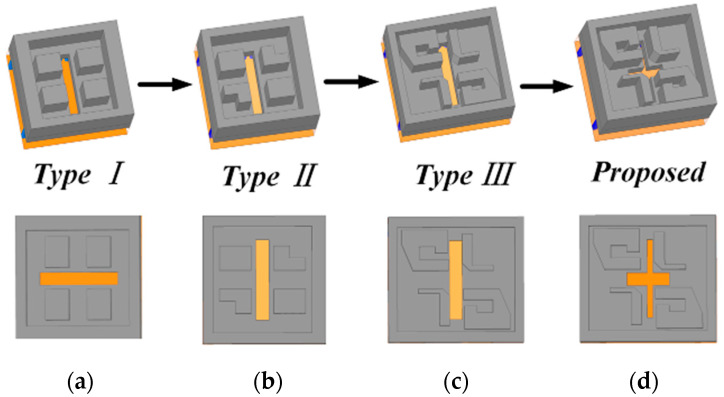
Design process of the proposed CP ME-dipole antenna element. (**a**) Type I: traditional LP ME-dipole. (**b**) Type II: introduce a suitable perturbation (**c**) Type III: introduce ring-shaped metal posts. (**d**) Proposed CP antenna element.

**Figure 3:**
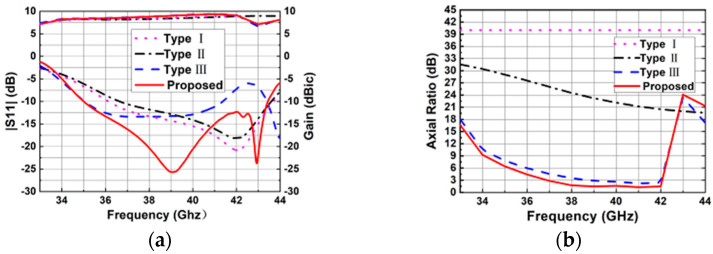
Performance comparisons of the three types of antenna elements. (**a**) Simulated |S11| and gains. (**b**) Simulated ARs.

The design processes of the proposed CP ME-dipole antenna element are shown in Figure 2. In Figure 2a, a traditional LP ME-dipole antenna is designed. It consists of two pairs of metal posts, a metal cavity and corresponding SIW feeding structures. In order to enhance the perturbation, the posts have been modified as shown in Figure 2b. To further enhance the 3 dB AR bandwidth, the four metal posts are divided into two groups which are symmetrical in the center. One group of posts is cut into an L shape, while the other group of posts is cut into a ring shape as shown in Figure 2c. Its simulated 3 dB AR bandwidth is 7.9% from 38.8 to 42.0 GHz as shown in Figure 3b. In the final proposed design, the rectangular coupling slot is modified as a cross-shaped slot with two different lengths and widths which yields a 13.2% 3 dB AR bandwidth from 36.9 to 42.1 GHz. As shown in Figure 3a, it can be seen that the impedance matching has been significantly improved by introducing the cross-shaped coupling slot.

**Figure 4:**
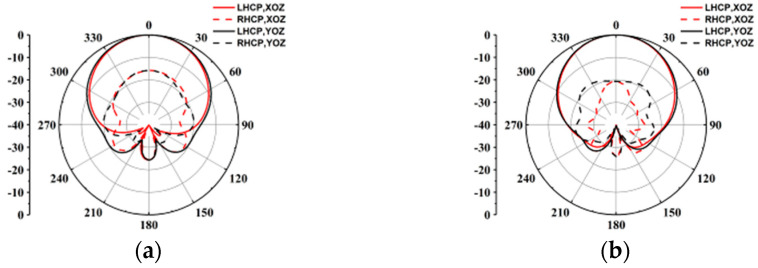
Simulated radiation patterns of the antenna element at (**a**) 37 GHz and (**b**) 40 GHz.

The simulated impedance bandwidth (|S11| ≤ −10 dB) of the proposed element is 21.4% from 35.0 to 43.4 GHz, while the simulated 3 dB AR bandwidth is 13.2% from 36.9 to 42.1 GHz. The simulated peak gain is 9.21 dBic at 40.5 GHz. The simulated results of the ME-dipole antenna element radiation patterns at 37 and 40 GHz are shown in Figure 4. The antenna element has stable gain, and the radiation patterns of the E-plane (yoz plane) and H-plane (xoz plane) are virtually equal.

**Figure 5:**
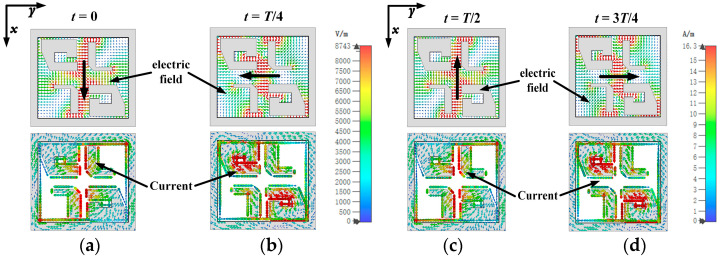
Simulated electric field and current distributions of the proposed antenna element at 39 GHz over a period of time. (**a**) t = 0. (**b**) t = T/4. (**c**) t = T/2. (**d**) t = 3T/4.

To better explain the operating mechanism of the circular polarization, the electric field distributions at four states (t = 0, T/4, T/2, 3T/4, where T is a time period) are shown in Figure 5. The black arrow represents the direction of the E-field vector at the corresponding time period, which, over a time period rotates in a clockwise manner, so a left-hand CP (LHCP) wave can be generated. As shown in Figure 5a, at the time t = 0, the fields between the metal posts arranged along the *y*-axis and the metal posts’ surface current are minimized. At t = T/4, the fields on the gap between the metal posts and the cavity walls and metal posts’ surface current dominate, as illustrated in Figure 5b. So, the electric dipole mode and magnetic dipole mode are excited at time t = T/4. As shown in Figure 5d, at time t = 3T/4, the electric dipole and magnetic dipole are excited again with opposite field direction compared to the mode at t = T/4. Therefore, it can be concluded that the ME-dipole mode is excited.

**Figure 6:**
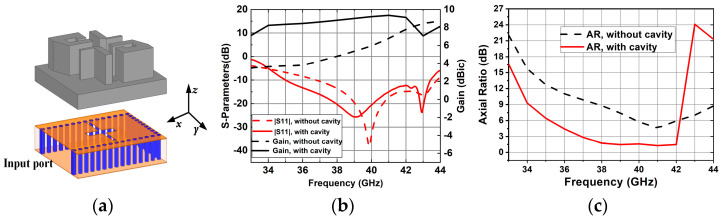
Geometry of the reference antenna element without a cavity. (**a**) 3D view. (**b**) Simulated |S11| and gains with and without cavity. (**c**) Simulated ARs with and without cavity.

**Figure 7:**
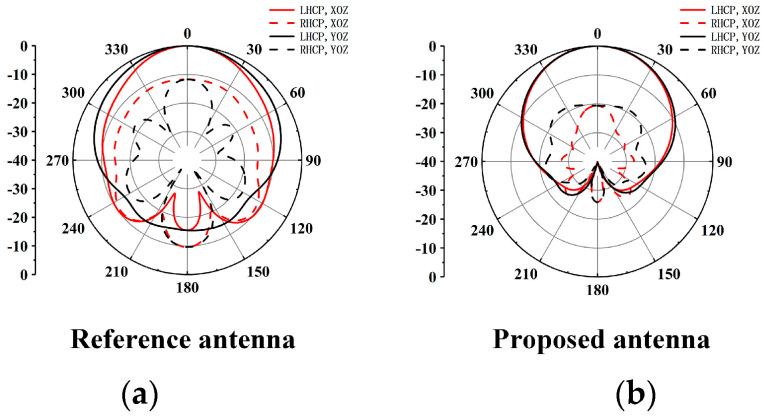
Comparisons of the simulated radiation patterns at 41 GHz. (**a**) the reference antenna element, (**b**) the proposed CP antenna element.

To illustrate the function of the metal wall, a reference antenna element without a cavity is designed. The dimensions of all the structural parameters of the reference antenna are the same as the proposed CP antenna element, as shown in Figure 6a. Figure 6b,c show the comparisons of simulated |S11|, gain and AR results between the proposed CP antenna element and the reference antenna element. It can be seen that the presence of the cavity improves the impedance matching of the antenna and broadens the 3 db AR bandwidth. The antenna gain is enhanced in the frequency range from 33 to 42 GHz. Comparisons of simulated radiation patterns of the reference antenna element and proposed CP antenna element are shown in Figure 7. Compared with the radiation pattern of the proposed CP antenna element, the uniformity of the radiation patterns in E-plane and H-plane of the reference antenna element without a cavity deteriorates. The metal cavity in the proposed design introduces two magnetic dipoles (the gap between the metal post and the cavity) along the *y*-axis, which enhances the characteristics of the ME-dipole of the antenna, so as to improve the uniformity of the radiation pattern in E-plane and H-plane. It can be seen that the metal cavity also decreases the back lobe level.

**Figure 8:**
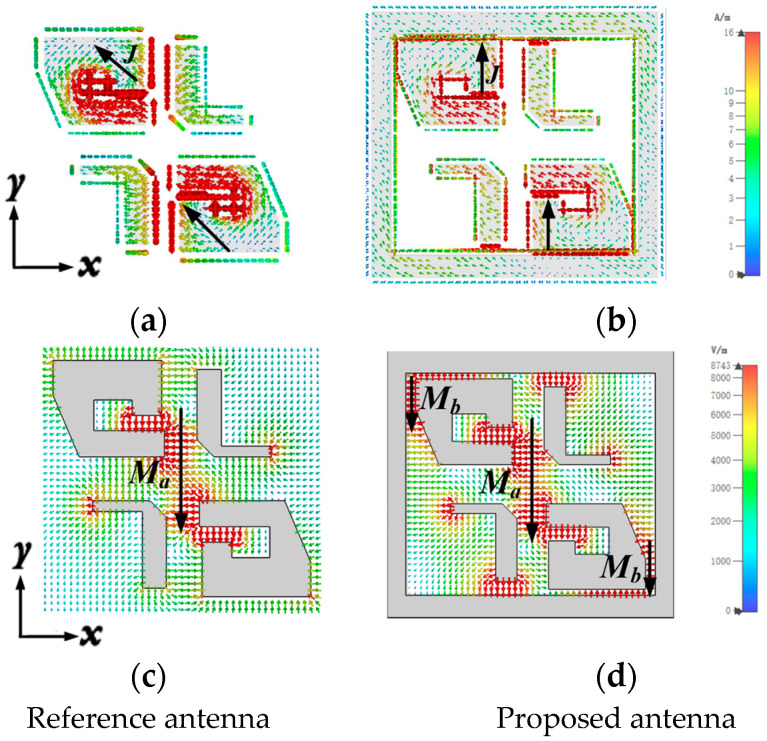
Surface current and electric field distribution between the reference antenna and the proposed antenna at 38 GHz. (**a**,**b**) Current distribution at t = 0. (**c**,**d**) Electric field distribution at t = T/4.

In order to explain the significant improvement of antenna circular polarization performance caused by loading a metal cavity, the surface current distribution and electric field distribution of antenna elements with or without cavities were simulated, as shown in Figure 8. According to electromagnetic field theory, when the electric dipole current is parallel to the magnetic dipole current, the electric field of the electric dipole and the magnetic dipole is orthogonal, and the condition of generating circularly polarized wave is satisfied. After loading the metal cavity, the surface current on the metal posts is better aligned along the *y*-axis, as shown in Figure 8a,b. As shown in Figure 8b,d, this is parallel to the magnetic dipole current when the magnetic dipole is excited at t = T/4, proving that the circular polarization performance of the antenna is greatly improved through the perturbation of the metal cavity. As shown in Figure 8d, it can be noted that the metal cavity also introduces a pair of magnetic dipoles between the metal posts and the metal cavity, enhancing the ME-dipole characteristics of the antenna.

**Figure 9:**
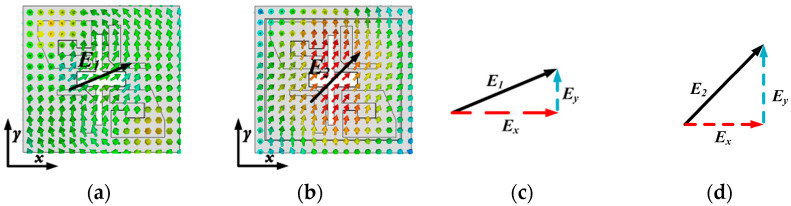
Simulated E-field distributions with the same phase at 38 GHz. (**a**) E-field distribution of the reference antenna. (**b**) E-field distribution of the proposed antenna. (**c**) Qualitative analysis of the E-field relationship of the reference antenna. (**d**) Qualitative analysis of the E-field relationship of the proposed antenna.

To delve deeper into the significant enhancement of the axial ratio (AR), we present the simulated electric field distributions of both the reference antenna and proposed antenna elements in Figure 9. According to the theory of electromagnetic fields, the generation of circularly polarized waves requires two linearly polarized components of constant amplitude and phase orthogonal. As depicted in Figure 9c,d, it is evident that upon loading the metallic cavity, there emerges an angular deviation between E1 and E2 at identical frequency points. This deviation predominantly stems from the reinforced electric field component along the *y*-axis subsequent to loading the metallic cavity. Consequently, through meticulous adjustments of the metallic posts, cross-slot, and metallic cavity dimensions, we can achieve two orthogonally linearly polarized components with a 90° phase difference at the cavity aperture.

#### 2.1.2. Parametric Studies

**Figure 10:**
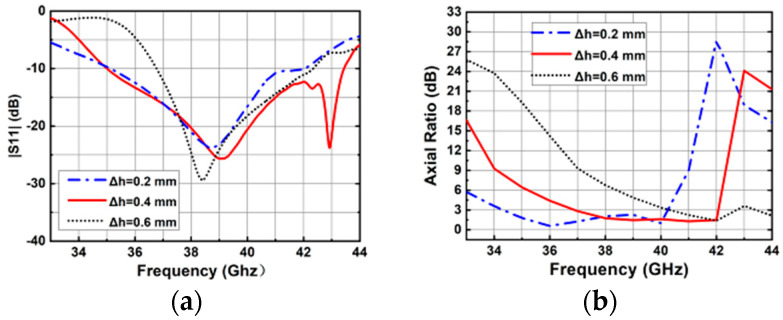
Simulated results of the proposed antenna element with different Δ*h* values. (**a**) |S11|. (**b**) ARs.

Studies of four important parameters, namely, Δ*h*, *w*_1_, *w*_5_, and *d*_2_, were performed for the proposed CP antenna element. In this design, the height of the cavity is 2.4 mm, the height of the metal post is 2 mm, and the height difference between them is set as the variable Δ*h*. Figure 10 shows the simulated results with different Δ*h*. As Δ*h* increases, the AR curves shift to a higher band, and the impedance matching is dramatically affected. Considering antenna matching and 3 db AR bandwidth, the optimized value of Δ*h* is finally selected as 0.4 mm.

**Figure 11:**
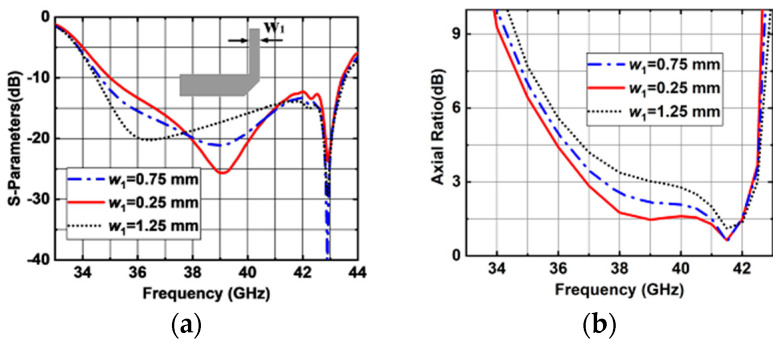
Simulated results of the proposed antenna element with different *w*_1_ values. (**a**) |S11|. (**b**) ARs.

Figure 11 shows the simulated results with different lengths of L-shaped metal posts (*w*_1_). The lower resonant frequency point shifts to a lower band as *w*_1_ increases, while the higher resonant frequency point remains virtually unchanged. However, the 3 dB AR bandwidth at the lower band decreases. The final value of *w*_1_ is optimized to 0.25 mm.

**Figure 12:**
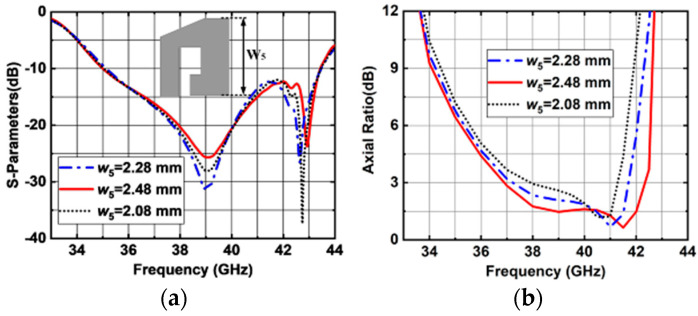
Simulated results of the proposed antenna element with different *w*_5_ values. (**a**) |S11|. (**b**) ARs.

**Figure 13:**
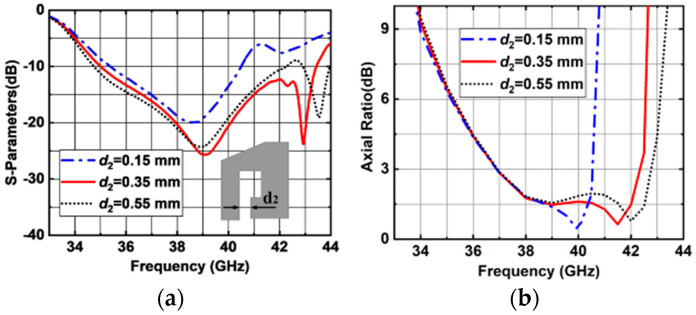
Simulated results of the proposed antenna element with different *d*_2_ values. (**a**) |S11|. (**b**) ARs.

The simulated |S11| and AR versus *w*_5_ are shown in Figure 12. When *w*_5_ varies, the impedance matching is slightly affected as shown in Figure 12a. The simulated results show that when *w*_5_ decreases, the AR performance deteriorates, as shown in Figure 12b. So, the optimized value of *w*_5_ is selected to be 2.48 mm. The simulated results show that upper and lower band AR could be optimized by the size of L-shaped and ring-shaped metal posts. It can be seen in Figure 13 that both the impedance matching, and AR performances are affected by the opening length of ring-shaped metal post (*d*_2_). As *d*_2_ increases from 0.15 to 0.55 mm, a wider 3 dB AR bandwidth can be achieved. However, the impedance matching deteriorates. Finally, the optimized value of *d*_2_ is selected to be 0.35 mm.

### 2.2. 4 × 4 Antenna Array Design

#### 2.2.1. Design of the 2 × 2 CP ME-Dipole Antenna Subarray

**Figure 14:**
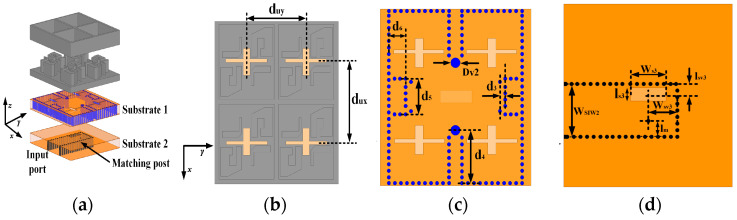
Geometry of the proposed 2 × 2 antenna subarray. (**a**) 3D exploded view. (**b**) Top view. (**c**) Substrate 1. (**d**) Substrate 2.

**Figure 15:**
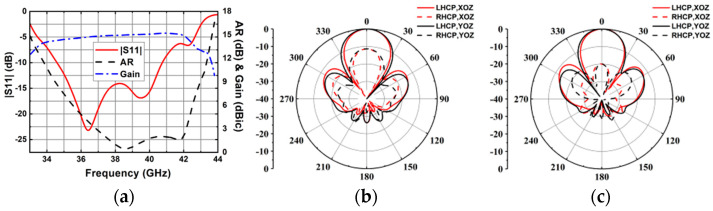
Simulated results of the 2 × 2 CP ME-dipole antenna subarray. (**a**) |S11|, gain, and AR. (**b**) Radiation pattern at 36 GHz. (**c**) Radiation pattern at 40 GHz.

Based on the proposed CP ME-dipole antenna element, a 2 × 2 CP antenna subarray was designed to obtain higher gain, as shown in Figure 14. In this subarray, a one-to-four wideband feeding network with two layers of 1.575 mm Rogers 5880 substrate is adopted. The energy is coupled from the rectangular slot on substrate 2 to four cross-shaped slots on substrate 1. The energy is equally distributed to the four coupling slots to excite four ME-dipole elements. By optimizing the double-layer four-port power divider, approximately equal power can be generated at the four output ports. The optimized dimensions of the 2 × 2 subarray are shown in Table 2. The simulated results for the 2 × 2 subarray are shown in Figure 15.

#### 2.2.2. Design of the 4 × 4 Antenna Array

**Figure 16:**
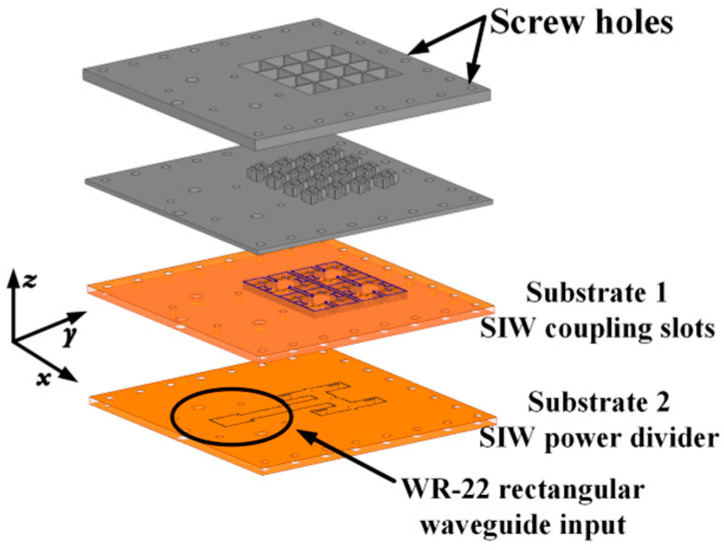
Geometry of the 4 × 4 CP antenna array.

**Figure 17:**
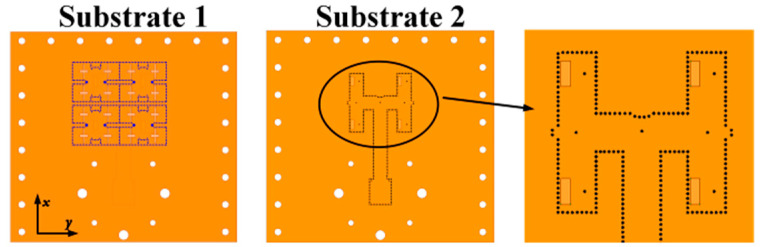
Geometric configurations of two-layer dielectric substrates.

Based on the 2 × 2 CP antenna subarray described above, a 4 × 4 antenna array was designed, with its geometry shown in Figure 16. The feeding network of the 4 × 4 antenna array is shown in Figure 17. The entire antenna array is arranged vertically. Substrate 2 contains a one-to-four SIW power divider and a standard WR-22 rectangular waveguide to SIW transition.

**Figure 18:**
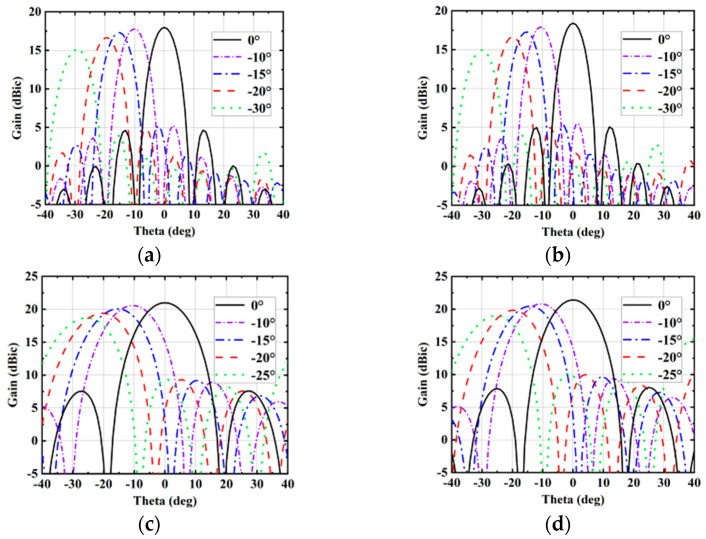
Radiation patterns of the yoz-plane of the proposed antenna array at various scanning angles. (**a**) 1 × 8 array at 38 GHz. (**b**) 1 × 8 array at 41 GHz. (**c**) 4 × 4 array at 38 GHz. (**d**) 4 × 4 array at 41 GHz.

In order to prove the beam scanning capability of the proposed antenna array, simulated results of 1 × 8 array and 4 × 4 array are given below. The beam scanning simulated performance of the 1 × 8 array at 38 and 41 GHz are shown in Figure 18a,b. At 38 and 41 GHz, the scan losses of 1 × 8 array are lower than 3.02 and 3.44 dBic, respectively. The 1 × 8 array can scan up to ±30°. The beam scanning simulated performance of the 4 × 4 array at 38 and 41 GHz are shown in Figure 18c,d. At 38 and 41 GHz, the scan losses of 4 × 4 array are lower than 2.33 and 2.39 dBic, respectively. The 4 × 4 array can scan up to ±25°. This proves that the proposed antenna array in this paper has relatively good beam scanning ability.

## 3. Experimental Results

**Figure 19:**
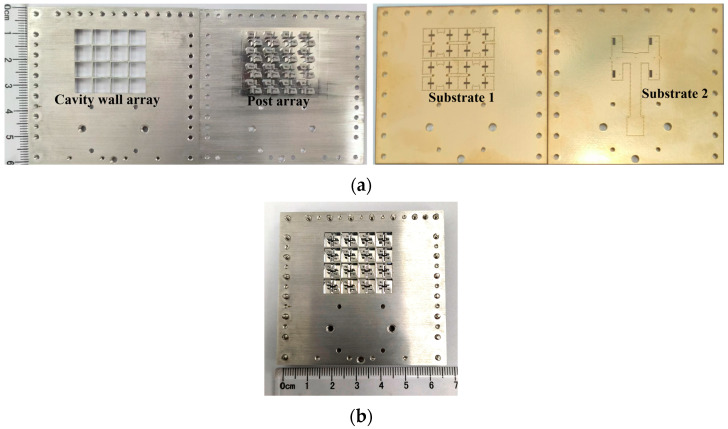
Fabricated 4 × 4 CP antenna array. (**a**) Disassembled drawing. (**b**) Assembly drawing of the prototype.

**Figure 20:**
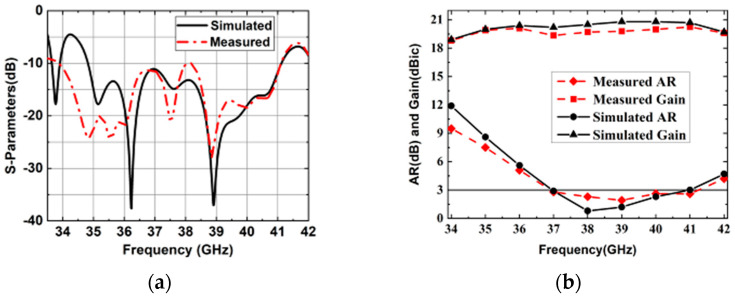
Simulated and measured results of the 4 × 4 CP antenna array. (**a**) |S11|. (**b**) ARs and gain.

The proposed 4 × 4 antenna array was fabricated using standard single-layer PCB technology, as shown in Figure 19. The screw holes were designed for assembly. The influences of the metal screws on the radiation performance of the array were included in the simulation. The overall dimensions of the antenna were 60.2 mm × 65.2 mm× 6.7 mm. Figure 20 shows the simulation and measurement results of this proposed 4 × 4 antenna array. It can be seen that the simulated and measured impedance matching the bandwidths of this antenna array are 16.8% (34.8–41.2 GHz) and 19.2% (33.9–41.1 GHz), respectively. The simulated and measured 3 db AR bandwidths are 10.3% (36.9–40.9 GHz) and 10.3% (37–41 GHz), respectively. The measured peak gain is 20.3 dBic at 41 GHz.

**Figure 21:**
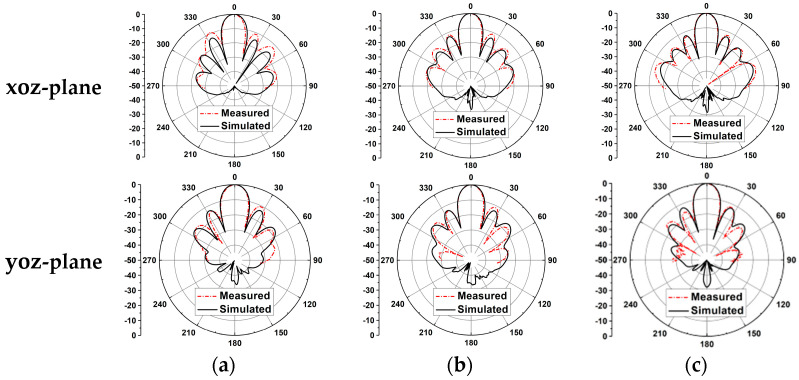
Simulated and measured radiation patterns of the 4 × 4 CP antenna array at (**a**) 37 GHz, (**b**) 39 GHz, and (**c**) 41 GHz.

**Figure 22:**
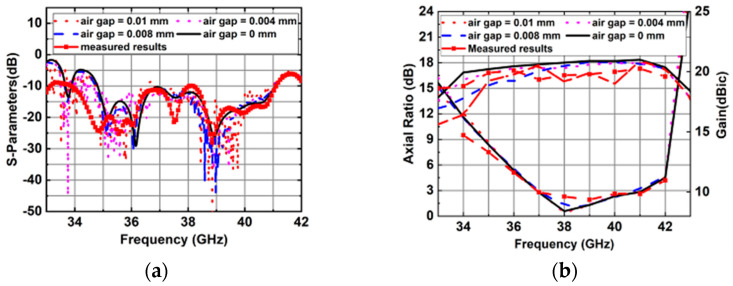
Simulated and measured results of the 4 × 4 CP antenna array with different thicknesses of the air gaps. (**a**) |S11|. (**b**) ARs and gain.

The measured radiation efficiency can be calculated by comparing the simulated directivity and measured gain. Then, the peak measured radiation efficiency of the 4 × 4 CP antenna array is 83.2% at 41 GHz. Figure 21 shows the simulated and measured radiation patterns of the 4 × 4 antenna array. It can be observed that the radiation patterns of the simulated and measured results are stable and consistent across the entire operating band. In the proposed CP antenna array, the aluminum plate and the two PCB substrates are fixed by screws; therefore, the effects of possible air gaps between substrate 1 and the aluminum plate should be considered. The simulated results of the 4 × 4 CP antenna array were considered. We took into account the possible air gaps between the PCB antenna array, with different air gap thicknesses are shown in Figure 22. Note that when the thickness of the air gaps reaches 0.008 and 0.01 mm, the impedance bandwidth deteriorates and the gains decrease at some frequencies, while the 3 db AR bandwidth remains largely unchanged. Also, the effect of the air gaps can be ignored when the thickness is 0.004 mm. This indicates that the possible air gaps can affect the performance of the assembled antenna array.

## 4. Discussion

Table 3 compares the proposed antenna array to previously reported CP MMW antenna arrays based on various parameters such as array configuration, thickness, impedance bandwidth, 3 db AR bandwidth and peak gain. Previous reports have highlighted that the majority of the 4 × 4 CP antenna arrays use microstrip radiating structures, resulting in peak gains less than 20 dBic. Thanks to the all-metal radiating structures, the proposed 4 × 4 antenna array obtains a high gain of 20.3 dBic. This work proposes a design method that can be utilized to develop a high-gain and low-cost MMW CP antenna array.

## 5. Conclusions

A high-gain SIW-fed MMW CP ME-dipole antenna array is proposed in this paper. A metal cavity is introduced to improve both the impedance and AR bandwidth, as well as to enhance the consistency of the E-plane and H-plane of the antenna. A 4 × 4 CP antenna array is designed, fabricated, and measured to validate the design concept. The measured results show that the CP antenna array achieves a wideband impedance bandwidth of 19.2%, a 3 db AR bandwidth of 10.3%, and a peak gain of 20.3 dBic. The measured results show that the antenna array has the advantages of high gain, high efficiency, etc. It demonstrates that the proposed high-gain CP antenna array is suitable for MMW communication.

## Figures and Tables

**Table 1:** Dimensions of the proposed antenna element (unit: mm).

Parameters	*w*_1_	*w*_2_	*w*_3_	*w*_4_	*w*_5_	*w*_6_	*w*_s1_	*w*_s2_	*w*_siw1_
Values	0.25	1.7	1.15	1.75	2.48	2.2	4.25	2.3	5
Parameters	*w*_c_	*l*_s1_	*l*_s2_	*l*_sv1_	*l*_sv2_	*l*_c_	*h*_w_	*h*_g_	*h*_p_
Values	5.7	0.3	0.7	3.4	2.5	6.4	2.4	1	2
Parameters	Δ*h*	*d*_1_	*d*_2_	*d*_p1_	*d*_p2_	*g*_x_	*g*_y_	*D*_v1_	*p*
Values	0.4	0.7	0.35	0.3	0.81	0.35	2.3	0.3	0.5

**Table 2:** Dimensions of the proposed 2 × 2 antenna subarray (unit: mm).

Parameters	*d*_3_	*d*_4_	*d*_5_	*d*_6_	*d*_uy_	*d*_ux_	*D*_v2_	*w*_s3_	*w*_sv3_
Values	0.45	4.15	2.8	1.43	6.15	6.82	0.8	2.7	2.2
Parameters	*w*_siw2_	*l*_s3_	*l*_sv3_	*l*_m_					
Values	4	1	0.8	1.2					

**Table 3:** Comparisons of MMW antenna arrays.

Ref.	No. of Element	Thickness (λ_0_)	*f*_0_ (GHz)	Type	Feeding Network	Imp. BW %	3 db AR BW %	Radiation Efficiency %	Peak Gain (dBic)
[3]	4 × 4	0.21	28.35	Slot	Microstrip line	28.6	14	>65	18.2
[4]	4 × 4	0.40	60	Helical	Microstrip line	22	20	n.a.	15.2
[6]	4 × 4	n.a.	60	Spiral	SIW	14.1	21.1	87.1	19.5
[9]	4 × 4	0.30	30.5	Parasitic patches	SIW	27.7	28.5	n.a.	17.85
[13]	4 × 4	0.15	45	ME-dipole	Microstrip line	>24.4	16	n.a.	18.8
[18]	4 × 4	0.02	28.63	ME-dipole	Microstrip line	21.83	5.9	n.a.	18.3
[22] *	8 × 8	2.20	31.5	ME-dipole	Air-filled waveguide	32.4	n.a.	72	27.1
[23]	4 × 8	2.19	90	ME-dipole	GWG	16.5	17	n.a.	23
This work	4 × 4	0.84	37.5	ME-dipole	SIW	19.2	10.3	83.2	20.3

λ_0_: free-space wavelength at center frequency. *: linearly polarized antenna array.

## Data Availability

Data are contained within the article.

## Abbreviations

The following abbreviations are used in this manuscript:

CP	Circularly polarized
ME-dipole	Magnetoelectric dipole
MMW	Millimeter-wave
SIW	Substrate-integrated waveguide
LP	Linearly polarized
LHCP	Left-hand circularly polarized
AR	Axial ratio

## References

[1] Deslandes D., Wu K. (2001). Integrated microstrip and rectangular waveguide in planar form. IEEE Microw. Compom. Lett..

[2] Li Y., Chen Z.N., Qing X., Zhang Z., Xu J., Feng Z. (2012). Axial ratio bandwidth enhancement of 60-GHz substrate integrated waveguide-fed circularly polarized LTCC antenna array. IEEE Trans. Antennas Propag..

[3] Wu J., Cheng Y.J., Fan Y. (2016). Millimeter-wave wideband high-efficiency circularly polarized planar array antenna. IEEE Trans. Antennas Propag..

[4] Liu C., Guo Y.-X., Bao X., Xiao S.-Q. (2012). 60-GHz LTCC integrated circularly polarized helical antenna array. IEEE Trans. Antennas Propag..

[5] Zhang T., Zhang Y., Cao L., Hong W., Wu K. (2015). Single-layer wideband circularly polarized patch antennas for Q-Band applications. IEEE Trans. Antennas Propag..

[6] Zhu Q., Ng K.-B., Chan C.H. (2017). Printed circularly polarized spiral antenna array for millimeter-wave applications. IEEE Trans. Antennas Propag..

[7] Zhang L., Wu K., Wong S.W., He Y., Chu P., Li W., Wang K.X., Gao S. (2020). Wideband high-efficiency circularly polarized SIW-fed s-dipole array for millimeter-wave applications. IEEE Trans. Antennas Propag..

[8] Zhang Y.-X., Jiao Y.-C., Zhang L. (2020). Wideband circularly polarized array antennas with sequential-rotation polarization grid and simplified full-SIW feeding networks. IEEE Trans. Antennas Propag..

[9] Zhu C., Xu G., Ding D., Wu J., Wang W., Huang Z.X., Wu X.L. (2021). Low-profile wideband millimeter-wave circularly polarized antenna with hexagonal parasitic patches. IEEE Antennas Wirel. Propag. Lett..

[10] Cheng Y., Dong Y. (2021). Wideband circularly polarized planar antenna array for 5G millimeter-wave applications. IEEE Trans. Antennas Propag..

[11] Sun G.-H., Wong H. (2022). Circularly polarized elliptical cavity-backed patch antenna array for millimeter-wave applications. IEEE Trans. Antennas Propag..

[12] Luk K.M., Wong H. (2006). A new wideband unidirectional antenna element. Int. J. Microw. Opt. Technol..

[13] Gan Z., Tu Z.-H., Xie Z.-M., Chu Q.-X., Yao Y. (2018). Compact wideband circularly polarized microstrip antenna array for 45 GHz application. IEEE Trans. Antennas Propag..

[14] Cao W., Wang Q., Qian Z., Shi S., Jin J., Ding K., Zhang B. (2018). Gain enhancement for wideband CP ME-dipole antenna by loading with spiral strip in Ku-band. IEEE Trans. Antennas Propag..

[15] Xiang L., Wu F., Yu C., Jiang Z.H., Yao Y., Hong W. (2022). A wideband circularly polarized magneto-electric dipole antenna array for millimeter-wave applications. IEEE Trans. Antennas Propag..

[16] Ding K., Li Y., Wu Y. (2022). Broadband circularly polarized magnetoelectric dipole antenna by loading parasitic loop. IEEE Trans. Antennas Propag..

[17] Tan Q., Fan K., Yu W., Yu Y., Luo G.Q. (2022). A broadband circularly polarized planar antenna array using magneto-electric dipole element with bent strips for Ka-band applications. IEEE Antennas Wirel. Propag. Lett..

[18] Xu J., Hong W., Jiang Z.H., Zhang H. (2020). Low-cost millimeter-wave circularly polarized planar integrated magneto-electric dipole and its arrays with low-profile feeding structures. IEEE Antennas Wirel. Propag. Lett..

[19] Li Y., Luk K.-M. (2016). A 60-GHz wideband circularly polarized aperture-coupled magneto-electric dipole antenna array. IEEE Trans. Antennas Propag..

[20] Ji Z., Sun G.-H., Wong H. (2022). A wideband circularly polarized complementary antenna for millimeter-wave applications. IEEE Trans. Antennas Propag..

[21] Feng B., Lai J., Chung K.L., Chen T.-Y., Liu Y., Sim C.-Y.-D. (2020). A compact wideband circularly polarized magneto-electric dipole antenna array for 5G millimeter-wave application. IEEE Trans. Antennas Propag..

[22] Sun F., Li Y., Ge L., Wang J. (2020). Millimeter-wave magneto-electric dipole antenna array with a self-supporting geometry for time-saving metallic 3-D printing. IEEE Trans. Antennas Propag..

[23] Cao J., Wang H., Mou S., Soothar P., Zhou J. (2019). An air cavity-fed circularly polarized magneto-electric dipole antenna array with gap waveguide technology for mm-wave applications. IEEE Trans. Antennas Propag..

